# Vitamin D_3_ receptor is highly expressed in Hodgkin’s lymphoma

**DOI:** 10.1186/1471-2407-12-215

**Published:** 2012-06-06

**Authors:** Christoph Renné, Alexander H Benz, Martin L Hansmann

**Affiliations:** 1 Senckenberg Institute of Pathology, University of Frankfurt, Theodor-Stern-Kai 7, 60596, Frankfurt am Main, Germany; 2 Department of Pathology, University of Giessen, Langhansstrasse 10, 35392, Gießen, Germany

## Abstract

**Background:**

Hodgkin lymphoma (HL) is one of the most frequent lymphoma in the western world. Despite a good overall prognosis, some patients suffer relapsing tumors which are difficult to cure. Over a long period Vitamin D has been shown to be a potential treatment for cancer. Vitamin D acts via the vitamin D receptor, a nuclear receptor, acting as an inducible transcription factor. We aimed to investigate the expression of vitamin D receptor as a possible diagnostic marker and potential therapeutic target in HL as well as in B-cell derived non-Hodgkin lymphoma (B-NHL).

**Methods:**

We used a panel of 193 formalin fixed tissues of lymphoma cases consisting of 55 cases of HL and 138 cases on several B-NHL entities.

**Results:**

Vitamin D receptor is strongly expressed in tumor cells of HL, regardless of the sub entity with an overall positivity of 80% of all HL cases. In contrast, only about 17% of the analyzed origin-NHL showed positivity for vitamin D receptor. The detection of nuclear localization of vitamin D receptor in the tumor cells of HL suggests activated status of the vitamin D receptor.

**Conclusions:**

Our study suggests VDR as a specific marker for tumor cells of HL, but not of B-NHL subtypes. Further, the observed nuclear localization suggests an activated receptor status in tumor cells of HL. Further investigations of mutational status and functional studies may shed some light in functional relevance of vitamin D receptor signaling in HL.

## Background

Hodgkin lymphoma (HL) is one of the most frequent lymphomas. Most of cases belong to the classical (c) subtype of HL (cHL), while only few percent of cases represent nodular lymphocyte predominant HL (NLPHL) [1]. The group of cHL is further subdivided into 4 distinct histological subgroups namely into nodular sclerosis (NS), mixed cellularity (MC), lymphocyte-depleted and lymphocyte-rich HL. In NLPHL, the tumor cells the so called lymphocyte predominant cells (LP), and also the tumor cells of cHL the Hodgkin and Reed-

Sternberg (HRS) cells account for a few percent of total tumor tissue the maiority of the cellular infiltrate comprises of T-lymphocytes, histiocytes, eosinophilic granulocytes and plasma cells [1]. All LP cells and mostly HRS cells are of B-cell origin [2-4]. Despite the HRS cells being mostly of B-cell origin they are lacking mostly of the B-cell expression program [5].

The genetic alterations involved in the pathogenesis of HL are still largely unknown. HRS cells in most if not all cases show numerical chromosome aberrations, amplifications and deletions of chromosomal subregions [6,7]. In addition, genetic alterations that contribute to constitutive NFκB activation in HRS cells have been identified [8-11]. For a large fraction of cases, infection of HRS-cell precursors with the Epstein-Barr virus (EBV) and expression of the viral latent membrane proteins (LMPs) 1 and 2a is an important pathogenetic event [12]. In HRS cells, many different signaling molecules and pathways such as Notch1, several receptor tyrosine kinases, the PI3K and MEK/ERK pathways and the transcription factors NFκB, STAT and AP-1 are aberrantly activated [13]. These pathways are frequently driven by autocrine as well as paracrine activation loops [14].

Vitamin D_3_ is a lipophilic molecule that belongs to the family of steroid hormones. Its endocrine functions in regulation of the calcium and phosphate utilization in bone metabolism are known for a long time. Apart from this endocrine character, newer studies added further functions for vitamin D_3_ especially in the pathogenesis of cancer. It has been shown that vitamin D_3_ is capable to modulate proliferation, differentiation, metastasis, invasion, angiogenesis and apoptosis via autocrine and paracrine mechanisms [15-18].

Vitamin D_3_ effects are mainly mediated by its receptor, the vitamin D receptor (VDR) to whom it binds in a high-affinity. Ligand binding is associated with conformational changes of the protein, allowing the interaction with dimerization partners and subsequent nuclear import [19]. VDR is a member of the superfamily of nuclear receptors for steroid hormones and acts as a heterodimer by binding to vitamin D responsive elements as a ligand inducible transcription factor [20]. Nuclear localization saves the active receptor complex from proteasomic degradation [19]. VDR expression can be induced by vitamin D_3_[21-23].

VDR is detectable by immunohistochemistry in dendritic cells [24]. In proliferating T-cells, VDR expression was shown to be high and correlating with the proliferative status, whereas VDR expression in B-cells is low in general [25,26]. Dendritic cells with tolerogenic properties can be induced by VDR [27,28].

Immunohistochemical studies revealed that VDR expression was detectable in most analyzed B-cell Non Hodgkin Lymphoma (B-NHL) cases; however expression was at very low levels compared with normal breast tissue as well as with breast carcinoma in which VDR has been shown to be of importance [29,30]. In diffuse large B-Cell lymphoma derived cell lines, treatment with vitamin D_3_ and a selective VDR-agonist reduced cell growth whereas again observed VDR expression was low [29].

In the present study, we investigated the expression of vitamin D receptor in a panel of HL and B-NHL in an immunohistochemical approach in order to identify a potential diagnostic marker and possible new therapeutical target for B-cell derived lymphomas.

## Methods

### Tissue samples

Tissue samples were retrieved from the files of the Department of Pathology of the University of Frankfurt and were originally submitted for diagnostic purposes. Cases were analyzed after approval was obtained from the University of Frankfurt School of Medicine Institutional Review Board for these studies. The study was performed according to the updated Helsinki declaration and informed consent was obtained from all patients.

### Antibodies

Primary antibodies were obtained from Santa Cruz (VDR, #sc13133, Santa Cruz, Heidelberg, Germany) and from Sigma (ß-Actin, Sigma, Deisenhofen, Germany). Secondary antibodies were purchased from Dako (Hamburg, Germany).

### Western blot analysis

Tissue lysates were prepared as described [31]. Proteins were separated by electrophoresis on 12,5% polyacrylamide gels containing 0,1% sodium dodecyl sulfate and were transferred to polyvinylidene difluoride membranes (Bio Rad, Munich, Germany) using tank blotting technique. Membranes were blocked with phosphate-buffered saline (PBS) containing 5% dry milk powder and 0,05% Tween20. Primary antibodies were used at dilutions of 1/5000 (for VDR) and 1:10000 (for ß-Actin) in PBS with 5% milk powder, and bound antibody was detected with the appropriate secondary HRP-coupled antibodies (Dako) in 5% milk supplemented with 0,05% Tween20 for 60 min. Signals were detected by using the ECL plus system (GE Healthcare, Munich, Germany), High Performance ECL chemiluminescence films (GE Healthcare) and Readymatic developer and fixer (Carestream Health Inc., NY, USA).

### Immunohistochemistry

Immunohistochemistry was performed with 5 μm sections of tissue of complete lymph node biopsies fixed in 5% buffered formalin embedded in paraffin. After dewaxing with xylene, antigen was retrieved by boiling the sections for 10 minutes in a microwave oven in 10 mM Na citrate at pH 6.0. Subsequently sections were blocked with BSA (5% in Tris buffered saline (TBS, Sigma). Sections were incubated for 12 hours with the primary antibody in at 4°C in TBS. Secondary, enzyme coupled mouse Ig-specific antibody (Dako) was incubated for 1 hour at room temperature. Staining was visualized with a catalyzed signal amplification Kit (K1500; Dako). For picture acquisition, an Olympus BX-51 microscope (Olympus, Hamburg, Germany) equipped with UPlanFL and PlanApo objective lenses (10x/0,3, 20x/0,5, and 40x/0,65) was used. Fotographs were processed with Adobe Photoshop 7.0 (Adobe, San Jose, CA).

Reactions lacking primary antibody did not show any noteworthy non specific background.

### Statistical analysis

Data analysis was performed using the GraphPad Prism 3.0 software (GraphPad, San Diego, CA). P-value in group comparison was determined using the two-sided Fisher's exact test. The criterion of significance was p < 0.05.

## Results and discussion

### VDR can be detected in formalin fixed tissue by immunohistochemistry

Like most cell types which are responsive to vitamin D_3_, also T-lymphocytes express constitutively VDR [32]. B-cells normally lack VDR, but receptor expression can be up regulated by specific stimuli [33-35]. Using RT-PCR, VDR expression could be demonstrated in naïve, germinal center and memory B-cells at low levels [36]. VDR expression in non activated B-cells in tonsils could only be detected after induction by vitamin D_3_ and interleukin 4 [25,36,37].

**Figure 1:**
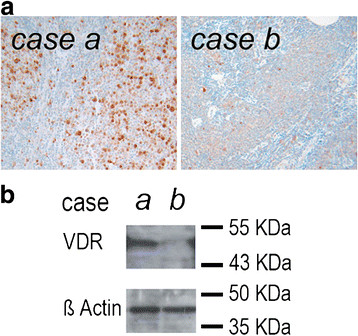
**Controls for the immunohistochemical staining of VDR.** (**a**) Immunohistochemical staining of VDR of two cases of B-NHL, both follicular lymphoma subtype: one case appearing rich in DC displays strong VDR positivity (case a) compared to another showing only few VDR positive DC (case b). (**b**) Frozen material of these cases was lysed and analyzed for VDR content by western blotting. In line with the immunohistochemical staining results, high VDR amount was detected in the DC rich case (case a), while a faint band was detected in the DC poor case (case b).

Using immunohistochemistry (IHC), VDR levels in B-cells in reactive lymph nodes or in hyperplastic tonsils were below detection limits (data not shown), while dendritic cells (DC), in line with previous data [24], stained positive as serving as an internal positive control (data not shown). High expression of VDR in DC served as a further control for the applied IHC method. To validate the IHC staining of VDR, Western blot analyses of B-NHL cases with high and low DC-content as obtained by IHC were performed using the corresponding frozen tissues. Signals for VDR were obtained at the expected molecular weight at around 48 kDa which appeared stronger in a DC-rich case of follicular lymphoma (FL). compared to a weaker signal in a FL-case with a low DC-count. (Figure 1)

### VDR is highly expressed in most HL cases but only in few B-NHL cases

Effects of sunlight, especially ultraviolet radiation regarding the risk of development of lymphoma, are discussed contradictorily [38-41]. Expression of VDR in B-cell lymphoma derived cell lines has been shown immunohistochemically long ago. However, expression levels of VDR in HL are still unknown [29].

We used immunhistochemistry to analyze a panel of B-cell lymphomas being composed of a total of 193 cases, namely 55 cases of HL (26 NSHL, 20 MCHL, 9 NLPHL), 138 cases of B-NHL (38 follicular lymphomas, 59 diffuse large B cell lymphomas, 10 mantle cell lymphomas, 5 marginal zone lymphomas, 6 Burkitt lymphomas, 10 primary mediastinal B cell lymphomas, 10 B cell chronic lymphocytic leukemias). A case was rated positive when more than 50% of the tumor cells displayed VDR-immunoreaction. This threshold was set in order to avoid underestimation of VDR expression in lymphoma cases with inhomogeneous staining due to possible fixation artifacts of the tissue. As seen before, DC also showed positivity in lymphoma cases and therefore served as an internal positive control.

**Figure 2:**
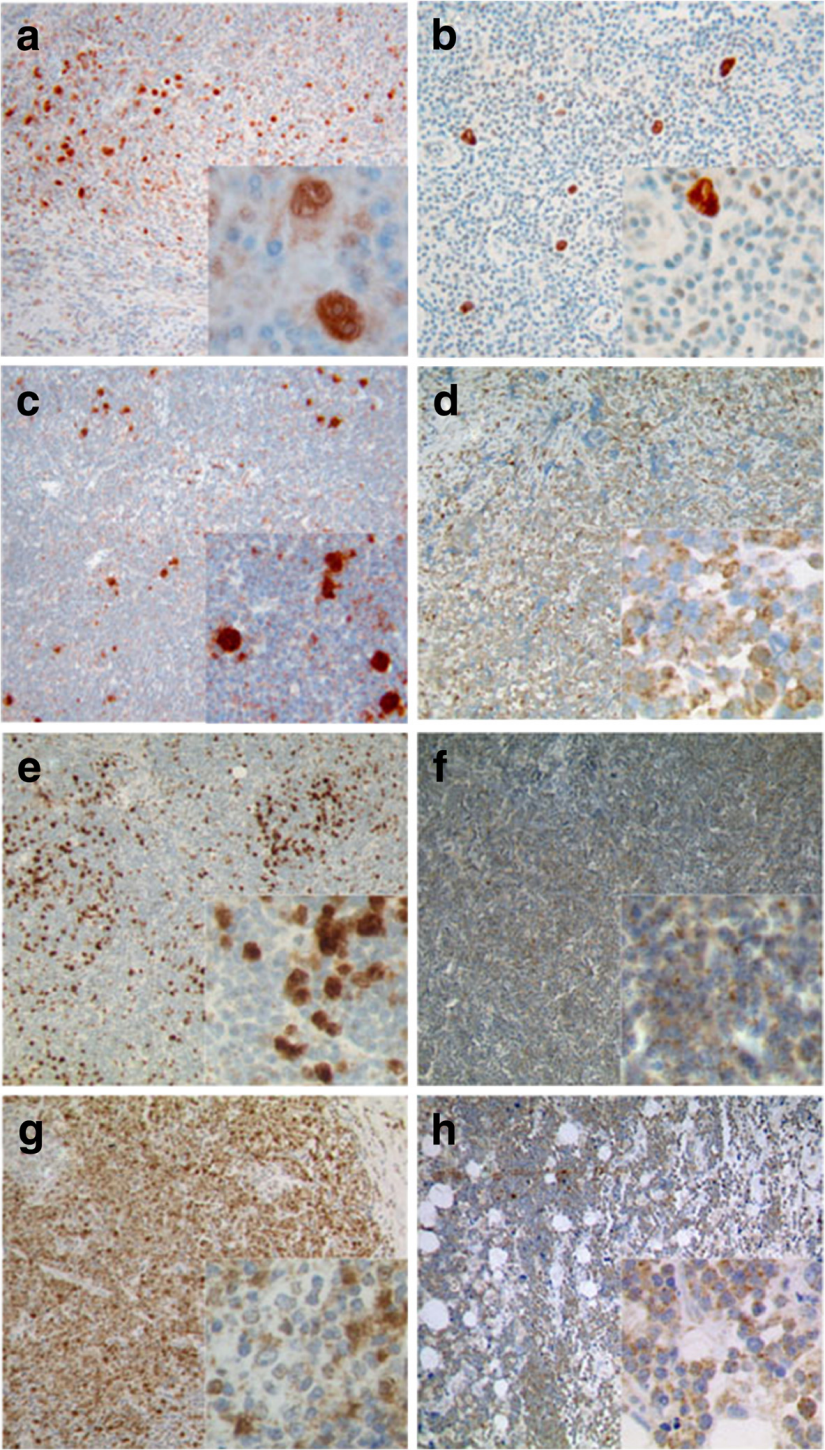
**Immunohistochemical detection of VDR in HL.** Exemplary cases of HL and B-NHL with expression of VDR are shown at low (10x) and high (40x, insert) magnification. The HL entities nodular sclerosing subtype (**a**), mixed cellularity subtype (**b**) and nodular lymphocyte predominant Hodgkin Lymphoma (**c**) are shown. Of B-NHL, samples of diffuse large B-cell lymphoma (**d**), follicular lymphoma (**e**), B-cell chronic lymphatic leukemia (**f**), marginal zone lymphoma (**g**) and Burkitt´s lymphoma (**h**) cases with staining reactions scored as positive are shown. In nodular sclerosing subtype of HL (A), also staining of activated T-cells and macrophages can be seen in the non neoplastic cellular background. Also in follicular lymphoma (E) macrophages staining strongly positive can be seen besides the tumor cells. B-NHL showed mostly cytoplasmatic positivity. Nuclear staining was found only in few cells of rare cases.

**Figure 3:**
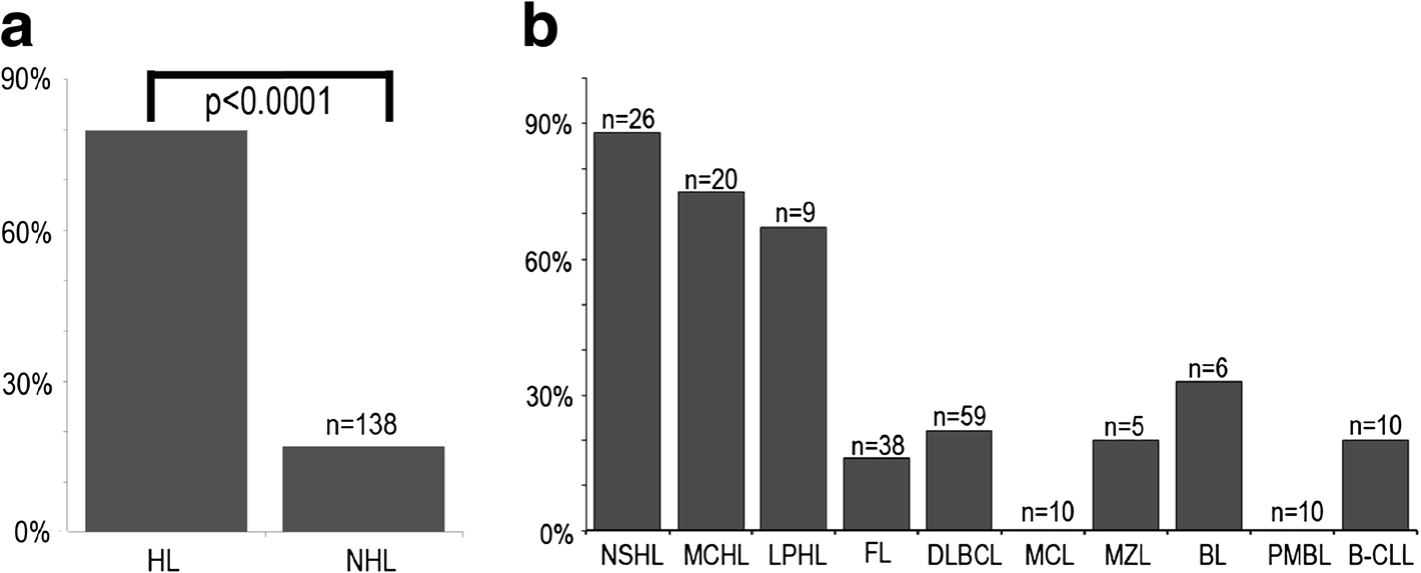
**Immunohistochemical detection of VDR in B-cell lymphoma.** 80% of HL showed strong nuclear positivity for VDR while positivity of B-NHL was only seen in 17.4% of cases (**a**). Among HL subtypes, VDR shows strongest positivity in classical subtypes NSHL (88.5%), followed by MCHL (75%). DLBCL with 22% and FL with 15.8% of positive cases are B-NHL subtypes with most prominent VDR reactivity. 33.3% of BL showed positivity for VDR, however only 6 cases have been analyzed (**b**).

VDR-positivity of tumor cells was observed in 80% (44/55) of HL cases but only in 17.4% (24/138) of total B-NHL entities tested (Figures 2 and 3). Using Fisher’s exact test on contingency tables, statistical analysis of VDR-positive cases revealed statistical significance between the group of HL and B-NHL (p < 0.0001).

Among the group of HL, the highest number of positive cases was found in NSHL with 88.5% (23/26) of cases, followed by MCHL with 75% (15/20) and NLPHL in 67.7% (6/9) of positive cases. The number of positive cases in B-NHL was clearly lower at 17.4% of cases (24/138). Of these, most positive cases were among DLBCL at 22% (13/59), FL at 15.8% (6/38) and BL 33.3% (2/6). (Figure 3b) However, only 6 BL could be analyzed so the number of positive cases might be different in a larger cohort. In the vast majority of HL B-NHL cases, HRS cells of HL and LP cells of NLPHL showed a cytoplasmic and also nuclear immunoreaction, whereas VDR was mainly detectable in the cytoplasm of the tumor cells B-NHL cases. Further, VDR staining intensity in tumor cells of HL was much stronger in general when compared to positive B-NHL cases. (Figure 2)

The strong VDR expression and nuclear localization in cHL as well as in NLPHL seems somehow unexpected as these lymphomas have different cellular origins and different marker profiles have been proven [1]. In a global gene expression analysis comparing NLPHL with other lymphoma types it was shown that NLPHL is despite the known differences very closely related to cHL. As cHL NLPHL is also characterized by a loss of marker molecules of B-cells. Both lymphoma entities show a strong constitutive NF-kB activity. Taken together it was shown that their global gene expression profiles differ only in few genes [42]. To go back to histological findings cHL and NLPHL both show a similar mixed cellular background of non neoplastic bystander cells which most likely provide the tumor cells of both entities with an analog pattern of paracrine stimuli.

Upon interaction with vitamin D_3_, retinoid-X-receptors are recruited to VDR, forming a receptor complex as heterodimerization partners. This complex migrates into the nucleus, binds to vitamin D responsive elements and activates transcription of dependent genes. Only few cell types have been described to show nuclear VDR without previous ligand induced activation [19,43]. Staining reactions for VDR in HL show mainly nuclear signals. Therefore it seems most likely that also in HRS cells VDR is ligand bound and activated, and thus detectable in the nucleus. (Figure 2a-c) Vitamin D3 activation by sunlight could not be taken as a definite ligand source, since influence of ultraviolet radiation has not been proven to have an influence on lymphoma risk [44]. Thus, activation of VDR raises the question of a possible endogenous source of ligand in tumor tissue. Given the typically low, subnanomolar plasma concentration of vitamin D_3_, availability of hormone in a suitable, effective concentration requires the presence of an exogenous cell source. Macrophages and monocytes on the one hand have been shown to express vitamin D_3_ and also been identified to express 1α-hydroxylase, the enzyme that is responsible activating vitamin D_3_ into its metabolic active form [45,46]. As these cell types are part the non neoplastic cellular infiltrate of HL, they might be a possible source of ligands acting in a paracrine manner.

About 40% of HL cases, mainly of MC subtype are infected with Epstein-Barr Virus (EBV) [1]. A recent study has shown that the EBV encoded protein EBNA3 is a possible binding partner of VDR, and that EBNA3 - VDR interaction blocks activation of VDR dependent gene expression, which protects EBV immortalized lymphoblast cell lines from vitamin D_3_ induced growth arrest and apoptosis [47]. As EBNA3 has not been detected in HRS cells, a relevant interaction of VDR with EBNA3 seems unlikely at least in HRS cells [12]. However, the interaction of EBNA3 with VDR in tumor cell progenitors of HL might be advantageous in earlier lymphoma development.

The VDR gene is located on chromosome 12q12-q14. Several single nucleotide polymorphisms of *VDR*, having possible influences on cancer pathogenesis, have been detected. Exemplarily, polymorphism rs2228570 results in a VDR variant which is three amino acids longer than its wild type and shows a lowered activity compared to the wild type [48]. Polymorphisms rs1544410 and rs731236 do not change VDR protein, but result in enhanced stability of VDR messenger RNA [49,50]. Association to DLBCL, and increased risk for FL upon exposure to ultraviolet radiation was reported for rs731236 [51,52]. As tumor cells in HL are very rare, micro dissection experiments would raise a possibility to elucidate genomic *vdr* mutation status in HL.

## Conclusions

In summary, this is the first study to report the strong expression of VDR in the vast majority of HL cases, while only low or none VDR expression was detected in normal, non neoplastic B-cells and all analyzed B-NHL. Nuclear localization of VDR suggests the active form of detected VDR in HL. As VDR activity seems to be of importance in B-NHL, VDR function in HL remains to be elucidated.

## Competing interests

All authors declare that they have no competing interests.

## Authors’ contributions

CR and AHB performed the experimental work. CR and MLH analyzed the data and drafted the manuscript. CR and MLH designed the study. All authors read and approved the final manuscript.

## Pre-publication history

The pre-publication history for this paper can be accessed here:

http://www.biomedcentral.com/1471-2407/12/215/prepub
